# Supplementary material for: Clinical features of talaromycosis in people living with HIV/AIDS (PWHA) and patients with anti-interferon-γ autoantibodies
Source: PLoS Negl Trop Dis. 2025 May 9;19(5):e0012992. doi: 10.1371/journal.pntd.0012992 (PMC12064013; doi:10.1371/journal.pntd.0012992)
Supplement: Attachment — Submitted filename: Cover letter_31052024.pdf [file pntd.0012992.s002.pdf]

FACULTY OF MEDICINE
CHIANG MAI UNIVERSITY

Division of Infectious Diseases and Tropical Medicine
Department of Internal Medicine Tel: 66-53-936739
Chiang Mai 50200, Thailand Fax: 66-53-894231

May 27, 2024

Dear editors, the PLOS Neglected Tropical Diseases

I hereby submit a revised manuscript entitled "Clinical features of talaromycosis in people living with HIV/AIDS (PLWHA) and patients with anti-interferon-γ autoantibodies" for your consideration for publication in PLOS Neglected Tropical Diseases as an original article. There are 2 copies of manuscripts: with and without highlighting.

The revisions include

1) Change the title from "Talaromycosis: differences between patients with anti-interferon-γ autoantibody and people living with HIV/AIDS (PLWHA)" to "Clinical features of talaromycosis in people living with HIV/AIDS (PLWHA) and patients with anti-interferon-γ autoantibodies"

2) Change the order of co-authors from "Narootchai Patanadamrongchai[1]*, Kawisara Krasaewes[2]*, Quanhathai Kaewpoowat [2,3], Saowaluck yasri[2], Antika Wongthanee1, Romanee Chaiwarith[2**] to "Kawisara Krasaewes[1], Narootchai Patanadamrongchai[2], Quanhathai Kaewpoowat[1,3], Saowaluck yasri[1], Antika Wongthanee[2], Romanee Chaiwarith[1*]

This is because Dr. Krasaewes collected an additional 171 PLWHA for this revised manuscript.

3) We included 235 PLWHA in this study (265 PLWHA were mentioned in the original version, but only 235 met the inclusion criteria).

FACULTY OF MEDICINE
CHIANG MAI UNIVERSITY

Division of Infectious Diseases and Tropical Medicine
Department of Internal Medicine Tel: 66-53-936739
Chiang Mai 50200, Thailand Fax: 66-53-894231

4) We used the "Instratext" to edit this manuscript and hope that this manuscript is better written in English.

5) Replace the new Tables 1 and 2

6) Replace the new Figure 1

7) Response to the editor

I have reviewed and agree with all suggestions by reviewers. In particular, I agree with the comment by reviewer #3 that the authors should include all 265 PLWHA over the study period, rather than randomly selected 64 patients as rationale for subgroup selection is unnecessary, unsubstantiated, and introduces bias. I also agree with the need to have the paper edited by an English-speaking medical writer.

Response: We included 235 PLWHA in this study (the original version mentioned 265 PLWHA, but only 235 met the inclusion criteria).

8) Response to reviewer 1

8.1 It is not clear whether all data were available for all patients and what was done with missing data (if any) - the authors only mention missing data in line 191

Response: Data from the hospital to which the patients were referred were not obtained to review. We only know the clinical characteristics and outcomes, but not the details of the treatment. We leave these data as missing values, i.e. treatment details. However, we have removed them from the manuscript to avoid confusion.

8.2 In Table 2, it is unclear what is meant with treatment and antifungals... is this treatment given on admission, or treatment given after *T. marneffei* infection was diagnosed?

Division of Infectious Diseases and Tropical Medicine
Department of Internal Medicine Tel: 66-53-936739
Chiang Mai 50200, Thailand Fax: 66-53-894231

Response: Change to "Treatment received" and "Medication" instead of "Treatment and antifungals" Treatment is at the time after diagnosis. We have added in line 123.

8.3 minor revision

8.3.1 line 76-77: were abscesses and nodules caused by *T. marneffei*, or other pathogens or unknown? The umbilicated lesions are basically pathognomonic for talaromycosis, the abscesses/nodules could be many other things

Response: We do not perform skin biopsies on all patients. The lesions described there are present in the patient at the time of diagnosis of talaromycosis. I agree that they could be caused by many other things. However, these lesions disappeared after treatment of the talaromycosis.

8.3.2 line 93-103: not sure if this extensive narrative on 2 patients is needed for this manuscript, probably not.

Response: We have removed these details.

8.3.3 the authors could consider to present the comparison with two other studies looking at HIV negative patients as a result rather than a discussion

Response: We believe these data should be in the discussion section as they are not the results of this study.

8.3.4 I don't think it's appropriate to thank patients who didn't consent and doctors who weren't aware of the study at the time in line 203-4.

Response: We have removed it.

FACULTY OF MEDICINE
CHIANG MAI UNIVERSITY

Division of Infectious Diseases and Tropical Medicine
Department of Internal Medicine Tel: 66-53-936739
Chiang Mai 50200, Thailand Fax: 66-53-894231

8.3.5 The introduction could do with some more information on burden of both talaromycosis and the described immunedeficiency. Some more clinical and epidemiological information on the immunedeficiency would also be helpful for the PLoS NTD readership- especially about presentation of other opportunistic infections. As talaromycosis appears to have a more pulmonary presentation in these patients, is this similar for TB and NTB mycobacteria? Does this disease mainly associate with lung infections? How does salmonellosis present, by BSI?

Response: Thank you for your suggestion. However, I would prefer not to go further into other opportunistic infections as we would like to focus on talaromycosis. In fact, we have done an analysis of the presentation of other OIs in the other preparatory manuscript and have not made it redundant.

8.3.6 There are some minor language errors throughout the manuscript.

Response: We have used “Instratext” as an editing tool for this manuscript and hope that this manuscript is better written in English.

9) Response to reviewer 2

9.1.1 The anti-interferon-gamma autoantibody is not clearly explained in the background knowledge.

Response: We have provided the references for the in-depth review of the knowledge on anti-interferon-gamma autoantibody

9.1.2 The detection method for anti-interferon-gamma autoantibody should be explained in detail.

Division of Infectious Diseases and Tropical Medicine
Department of Internal Medicine Tel: 66-53-936739
Chiang Mai 50200, Thailand Fax: 66-53-894231

Response: This has already been mentioned in line 60.

9.1.3 Is it also possible for HIV/AIDS patients to have anti-interferon-gamma autoantibody? Should these patients be excluded?

Response: We have not checked anti-IFN-gamma antibodies in patients who had CD4 < 200 cells/μg.

9.1.4 A flowchart explaining the process of screening the thirty-two patients with antiIFN- γ AAb should be provided.

Response: Only those who met the inclusion criteria were included in the analysis.

9.1.5 The expression of CD4+T lymphocytes is inaccurate.

Response: We have corrected to CD4 cells

9.1.6 The word 'Gou' should be 'Guo' on page 13, line 180.

Response: We have corrected this

9.1.7 This study shows a lower patient mortality rate than the literature generally, but the reasons have not been explained.

Response: We have amended the sentences as follows: “Our earlier study showed that the mortality rate in HIV-uninfected patients was 20.7%. However, the exact proportion of patients with anti-IFN-γ-AAbs was not known (Kawila et al. 2013).” Lines 228-229.

9.1.8 The paper presentation requires significant improvement. In its present form, it is hard to read. There are many typos, syntax and grammatical errors that it is very difficult to enumerate them all.

FACULTY OF MEDICINE
CHIANG MAI UNIVERSITY

Division of Infectious Diseases and Tropical Medicine
Department of Internal Medicine Tel: 66-53-936739
Chiang Mai 50200, Thailand Fax: 66-53-894231

Responses: We have corrected them

9.1.9 The methods description in your Abstract section is not clear. Please update it.

9.1.10 The following sentence should be added in Data Analysis section.

"Statistical significance was set as a $P \leq 0.05$ for all analyses."

Response: We have added it in line 78-79.

9.1.11 The quantitative variables should be described with median rather than the mean.

Response: This depends on the distribution of the data. If they are normally distributed, we use mean and SD, if they are not normally distributed, we use median and IQR.

9.1.12 "PLWHA" should be used when the term "people living with HIV/AIDS" appeared in second time.

Response: We have removed it.

9.1.13 The following description (Line 83-84) is inconsistent with the content of Table 2. Please check it carefully.

"Abnormal chest radiograph was less common in patients with anti-IFN- γ AAbs."

Response: We have corrected this

9.1.14 Some terms that appeared in your Results section cannot be found in any Tables and Figures. For example: leukocytosis, paravertebral abscess, subcutaneous abscess. Please check it carefully.

Response: We have corrected this

9.1.15 Please unify the writing of digital that in your Results section and Tables.

Response: We have corrected them

FACULTY OF MEDICINE
CHIANG MAI UNIVERSITY

Division of Infectious Diseases and Tropical Medicine
Department of Internal Medicine Tel: 66-53-936739
Chiang Mai 50200, Thailand Fax: 66-53-894231

9.1.16 The following descriptions (Line 93-103) in Treatment section are not the part of your study results. Please remove it and update this section.

Response: We have removed them

10) **Response to reviewer 3**

Major Revisions

10.1 Methodology and selection of PLWHA – it is unclear from the methods section why 64 patients were selected from the underlying population of 265 PLWHA. Were they matched with the auto-immune group and what characteristics were used for matching? If they were not matched, what was the purpose of selecting 64 patients? This is a descriptive paper rather than an analytic paper, my suggestion here would be to include all 265 patients in the analysis rather than randomly selecting 64. A description of the entire PLWHA cohort during the study period would be of most value in the current paper.

10.2 Materials and methods - overall this section does not contain enough detail, particularly in justifying the choice of 64 patients randomly selected from the underlying PLWHA cohort

Response to 10.1 and 10.2

We included 235 PLWHA in this study (the original version mentioned 265 PLWHA, but only 235 met the inclusion criteria).

10.3 Discussion section: This section should emphasize what this manuscript adds to the overall literature on this topic. This rationale is not evident in the discussion section. Authors should emphasize in the early sentences of this section what this manuscript adds to the current literature.

Division of Infectious Diseases and Tropical Medicine
Department of Internal Medicine Tel: 66-53-936739
Chiang Mai 50200, Thailand Fax: 66-53-894231

Response: We have reworded this sentence in lines 230-231.

10.4 Minor revisions

10.4.1 Grammer, syntax and typographical errors– This manuscript would benefit from the review of a medical writer or medial editor. While the errors in English syntax and grammar, as well as typographical errors are minor, however they are numerous throughout the manuscript. Correcting these errors would improve the readability of the manuscript and I believe are essential prior to publication

Response: We have used “Instratext” as an editing tool for this manuscript and hope that this manuscript is better written in English.

10.1.2 Table 1: Row headings need to include what the value in brackets refers to. Ie for previous/concurrent OIs, the values within parentheses presumably refer to column percentages.

Response: We have added this sentence under Table 1.

“Data are presented in number (%), mean (standard deviation) or median (IQR)”

10.1.3 Table 1: Median (IQR) days from symptom to diagnosis, for the autoimmune column there should be a comma between 14 and 75)

Response: We have corrected it.

10.1.4 Fig 1: The authors note that there is not "statistically significant [difference]" between the two groups, however the Fig does seem to demonstrate the the KM curves are separate and

FACULTY OF MEDICINE
CHIANG MAI UNIVERSITY

Division of Infectious Diseases and Tropical Medicine
Department of Internal Medicine Tel: 66-53-936739
Chiang Mai 50200, Thailand Fax: 66-53-894231

do not cross. Is it correct that there are no differences in mortality between the two groups?

Also note typographic error in the Mortality section of the results ("statistically significant...")

Response: The KM curve shows the point estimate, not the two 95%CIs, which overlap. We have added below the figure legend.

Sincerely,

Romanee Chaiwarith

Romanee Chaiwarith, MD, MHS.
