# Supplementary material for: Clinical features of talaromycosis in people living with HIV/AIDS (PWHA) and patients with anti-interferon-γ autoantibodies
Source: PLoS Negl Trop Dis. 2025 May 9;19(5):e0012992. doi: 10.1371/journal.pntd.0012992 (PMC12064013; doi:10.1371/journal.pntd.0012992)
Supplement: Attachment — Submitted filename: Response to reviewer_19082024.docx [file pntd.0012992.s003.docx]

**Reviewer's Responses to Questions**

**Reviewer #1**

1. Line 127-8: Correct to: "In three PLWHA, physicians missed the diagnosis of talaromycosis but these three PLWHA received..."
2. Line 128-9: What do the authors mean with: "Ten PLWHA survived at the time this study was conducted." - seems a very low number out of a group of 235 patients, typo - or am I misunderstanding something?

**Response to Q1 and Q2:** We have made the correction as now in lines 133-146 as follows.

Two patients with anti-IFN- γ-AAbs missed the diagnosis of talaromycosis and were not treated (number 1 and 2 in Table 3). Thirty patients received treatment; 29 were treated with itraconazole initially (96.7%), one patient who was treated with amphotericin B and then itraconazole died of hospital-acquired pneumonia after clinical condition improved following treatment with amphotericin B and then itraconazole for talaromycosis (number 3 in Table 3).

Nine PLHWA died before a diagnosis of talaromycosis was made; 6 received empirical amphotericin B treatment (number 4-9 in Table 3) and 3 were not treated (number 10-12 in Table 3). Of the 217 remaining patients, 9 patients were transferred and one of them died (number 13 in Table 3), 4 missed the diagnosis of talaromycosis and were not treated and died (number 14-17 in Table 3), 3 missed the diagnosis of talaromycosis and received fluconazole as primary prophylaxis and survived. Two hundred and sixteen received treatment; 51 received itraconazole initially (53.3%) and no one died, 97 received amphotericin B initially and 22 of them died (18-39 in Table 3). (Fig. 1).

3. Line 130: Does it require explanation that the recommended treatment of AmphoB is consistently not used in patients with anti IFN-gamma abs? Is this per guidelines? The proportion of PLWHA receiving amphoB is also very low. Any mortality differences observed between the amphoB and itra only groups?

**Response:** The explanation is added in lines 240-258 as follows

Itraconazole has a low minimum inhibitory concentration (MIC) against T. marneffei (Sekhon et al. 1993; Supparatpinyo et al. 1993) and has been shown to be effective in the initial treatment of talaromycosis with mild disease. (Supparatpinyo et al. 1992; Supparatpinyo et al. 1992) The archived guidelines (through October 22, 2019) for the prevention and treatment of opportunistic infections in HIV-infected adults and adolescents from the Centers for Disease Control and Prevention, the National Institutes of Health, and the HIV Medicine Association of the Infectious Diseases Society of America suggest that initial treatment with itraconazole is the preferred therapy for patients with mild disease. (CDC, NIH, HIVMA, IDSA 2019) However, since the November 21, 2019, itraconazole is no longer recommended as induction therapy for talaromycosis, as the results of IVAP trial in PLWHA, which showed the superiority of amphotericin B over itraconazole in the induction phase, regardless of disease severity and faster fungal clearance in blood culture. (Le T et al. 2017; CDC, NIH, HIVMA, IDSA 2019) Whether similar results apply to other populations, including patients with anti-IFN- γ-AAbs, and whether faster fungal clearance may influence clinical outcomes in patients with anti-IFN- γ-Abs remains to be determined. Not surprisingly, all but one patient with anti-IFN- γ-AAbs initially received itraconazole because they were not ill enough to be hospitalized in a hospital with limited admission capacity and were willing to receive oral therapy and be closely monitored in the outpatient clinic as they were able to return to work.

4. Line 222 and further: should it just be "mortality", rather than "mortality rate" (5 times)

**Response:** The mortality is death. The mortality rate is the calculation of the number of patients who died divided by the total number of patients diagnosed with talaromycosis. Therefore, I would prefer to use mortality rate and not mortality.

**Reviewer #2:**

1. In Table 2, all individuals with anti-IFN- γ-AAbs used itraconazole. So, what prevents patients from choosing AMB？

**Response:** The explanation is added in lines 240-258 as follows

Itraconazole has a low minimum inhibitory concentration (MIC) against T. marneffei (Sekhon et al. 1993; Supparatpinyo et al. 1993) and has been shown to be effective in the initial treatment of talaromycosis with mild disease. (Supparatpinyo et al. 1992; Supparatpinyo et al. 1992) The archived guidelines (through October 22, 2019) for the prevention and treatment of opportunistic infections in HIV-infected adults and adolescents from the Centers for Disease Control and Prevention, the National Institutes of Health, and the HIV Medicine Association of the Infectious Diseases Society of America suggest that initial treatment with itraconazole is the preferred therapy for patients with mild disease. (CDC, NIH, HIVMA, IDSA 2019) However, since the November 21, 2019, itraconazole is no longer recommended as induction therapy for talaromycosis, as the results of IVAP trial in PLWHA, which showed the superiority of amphotericin B over itraconazole in the induction phase, regardless of disease severity and faster fungal clearance in blood culture. (Le T et al. 2017; CDC, NIH, HIVMA, IDSA 2019) Whether similar results apply to other populations, including patients with anti-IFN- γ-AAbs, and whether faster fungal clearance may influence clinical outcomes in patients with anti-IFN- γ-Abs remains to be determined. Not surprisingly, all but one patient with anti-IFN- γ-AAbs initially received itraconazole because they were not ill enough to be hospitalized in a hospital with limited admission capacity and were willing to receive oral therapy and be closely monitored in the outpatient clinic as they were able to return to work.

2. Does the mortality rate include the untreated population? How to determine the mortality rate of patients who have been transferred to other hospitals

**Response:** The mortality rate is calculated by the number of patients who died divided by the total number of patients. The vital status for those who have been transferred to other hospitals was obtained from the Thai civil registry. It already existed in the method part of the study in line 68.

3. Are the deaths mainly caused by talaromycosis or other comorbidities?

**Response:** The cause of death was described in new table 3.

**Reviewer #3:**

1. The authors have corrected the major methodological challenge with the original submission, which was around selection of the HIV patients. This has now been rectified with inclusion of all 235 HIV patients. This vastly strengthens the conclusions of the paper, and I note some of the results have indeed changed with inclusion of the larger HIV cohort. However I note that Table 2 still only discusses 64 HIV patients - why is this?

**Response:** Thank you for your comment. That was the error. We have checked and corrected from 64 to 235 in table 2

2. Line 128, I don't understand what this sentence means " Ten PLWHA survived at the time this study was conducted." This seems like a high mortality rate among HIV patients if correct.

**Response:** We have corrected the correction as now in lines 133-146 as follows.

Two patients with anti-IFN- γ-AAbs missed the diagnosis of talaromycosis and were not treated (number 1 and 2 in Table 3). Thirty patients received treatment; 29 were treated with itraconazole initially (96.7%), one patient who was treated with amphotericin B and then itraconazole died of hospital-acquired pneumonia after clinical condition improved following treatment with amphotericin B and then itraconazole for talaromycosis (number 3 in Table 3).

Nine PLHWA died before a diagnosis of talaromycosis was made; 6 received empirical amphotericin B treatment (number 4-9 in Table 3) and 3 were not treated (number 10-12 in Table 3). Of the 217 remaining patients, 9 patients were transferred and one of them died (number 13 in Table 3), 4 missed the diagnosis of talaromycosis and were not treated and died (number 14-17 in Table 3), 3 missed the diagnosis of talaromycosis and received fluconazole as primary prophylaxis and survived. Two hundred and sixteen received treatment; 51 received itraconazole initially (53.3%) and no one died, 97 received amphotericin B initially and 22 of them died (18-39 in Table 3). (Figure 1).

3. Line 130 - need to specify that 102/216 refers to HIV patients (if this is the case)

**Response:** yes, they PLWHA. However, we have deleted this sentence and described more explanation now in lines 133-146.

4. Line 148 - could phrase this better ie "This study has shown that the clinical manifestations of talaromycosis varies depending on the immunocompromising condition of the host."

**Response:** The sentence is re-phrase as shown in lines 181-183.

5. Line 234 - check this paragraph. Lists first limitation then jumps to third limitation. I also think the limitiation section in general could do with some further thought - i.e this is a retrospective studies which introduces bias, missing data, unmeasured confounders, etc

**Response:** The second limitation has been added on lines 272-275.

6. line 114 - needs a full stop.

**Response:** The full stop is added.

7. Table 2 and Table 3 - it is not clear what the values mean for some of the rows. Need to specify what the figures in brackets are referring to.

**Response:** We have added this phrase under tables 2 and 3

*Data are presented in number (%), mean ± standard deviation, and median (IQR) as appropriate

8. It is unique and valuable that this study describes the clinical differences of talaromycosis patients between anti IFN- γ AAbs and PLWHA. As a result of this study, it is possible to gain an understanding of the characteristics and prognosis of talaromycosis in two populations. The shortcoming is that the background has not yet clearly explained the differences in treatment and prognosis between the two groups. This makes the data on treatment and prognosis appear abrupt. Furthermore, no in-depth discussion or analysis of some meaningful differences was provided in the discussion section.

**Response:** Thank you for your suggestion. However, I think the background to the reasons for doing this study has already been covered. I would prefer to leave them as they are. We have added some suggestions for further research on lines 252-254 in the discussion section.

9. The three reviewers of the manuscript had raised a lot of detailed questions. However, the authors' reply was not very satisfactory, especially some key questions. For example, the reviewers mentioned the need for a clear flowchart, the background and detection details of anti-interferon-gamma autoantibody, the lack of detailed description of the methodology, and so on, which have not been substantially modified in the manuscript.

**Response:** The flow chart of the study participant has been added in figure 1. The methodology of anti-interferon-gamma autoantibody has been added on lines 70-75.

10. There are still some problems with the manuscript, such as "Demographic data, clinical characteristics, laboratory findings, treatment and treatment results were recorded" mentioned by the authors, so where does the information come from, how to obtain it, and how to ensure authenticity, reliability and standardization? More details need to be provided.

**Response:** All the data are retrieved from existing hard copy of medical record or electronic medical record. The vital status of the patients who referred or transferred to other hospital retrieved from Thai civil registry. The information has been added on lines 66.

11. The authors mentioned a total of 38 deaths, so do you analyze the specific causes of their deaths?

**Response:** The details of the causes of 39 deaths have been added in a new Table 3.

12. With regard to the limitations, the author did not seriously evaluate its potential impact on this study and the direction of future research that needs to be improved. In addition, the second point of limitation is missing.
    **Response:** The second limitation has been addressed on lines 304-307. In the discussion, we also have added the further study in needs for patients with anti-IFN- γ-AAbs on lines 252-254.
13. The discussion part is limited to superficial comparison and lack of conciseness. In addition, it is not clear what this study has added to the previous literature, what scientific significance it has, and what kind of impact it has on clinical practice.

**Response:** To our knowledge, this is the first study to directly compare the clinical features of talaromycosis between PLWHA and patients with anti-IFN- γ AAbs. Previous studies make the comparison between PLWHA and non-AIDS patients, or present only PLWHA or only in patients with anti-IFN- γ AAbs, but do not make the comparison in the same study, in which study population are in the similar environment, and medical care. I think this represents a scientific value in this area. The discussion has aligned with the objectives of this study.
