# Supplementary material for: Clinical features of talaromycosis in people living with HIV/AIDS (PWHA) and patients with anti-interferon-γ autoantibodies
Source: PLoS Negl Trop Dis. 2025 May 9;19(5):e0012992. doi: 10.1371/journal.pntd.0012992 (PMC12064013; doi:10.1371/journal.pntd.0012992)
Supplement: Attachment — Submitted filename: Response to reviewer_28012025.docx [file pntd.0012992.s004.docx]

**Reviewer's Responses to Questions**

The line numbers in the responses refer to a track-changed version.

1. Briefly describe how the assay was modified here.

**Response:** The description is provided on lines 75-80 as follows:

The screening test for antibodies to IFN-γ was performed using an enzyme-linked immunosorbent assay (ELISA), which was modified from the methods previously described by Tang et al. (Tang et al. 2010) In brief, 2% skimmed milk in phosphate-buffer saline (PBS) was used in blocking buffer and diluent, replacing the 5% normal goat serum used in the original method. Additionally, o-phenylenediamine dihydrochloride (OPD) was utilized as the substrate instead of tetramethylbenzidine (TMB), as described by Tang et al. (Tang et al. 2010) AThe confirmatory test for antibodies against IFN-γ was then performed using an inhibition ELISAassay. The details of the methods were described in the study by Wongkulab et al. (Wongkulab et al. 2013)

2. Why were both softwares STATA and R used?

**Response**: Since the initial submission of the manuscript, we originally used STATA software for data analysis. However, after the reviewers requested the inclusion of additional patients in the study, we have since conducted the analysis using R software. As a result, we have removed the mention of STATA from the sentences on line 96.

3. Revise this. These are not different.

**Response**: The sentence on line 140 is deleted.

4. Clarify that this patient survive without receiving treatment

**Response:** The sentence clearly indicates that all three patients survived; therefore, no changes have been made to it. However, we have rewritten the paragraph from lines 143–166 to align with the removal of Table 3.

5. Data from the multiple variable regression should be presented as a separate paper

**Response**: We deleted them from the paragraph (lines 175-177).

6. Please remove Table 3 as it is not informative, and instead provide a summary of the causes of death as text.

**Response:** Table 3 is removed. The cause of death is described on lines 177-182.

7. Please shorten this paragraph and move this paragraph along with Figure 4 in the Results section. Do not present in the Discussion.

Response

1. The first paragraph in the discussion is removed from the manuscript (lines 221-232)
2. Lines 233-237 in the first paragraph of the discussion is moved to the result section under the subheadings “Comparisons of clinical characteristics of talaromycosis in patients with anti-interferon-γ autoantibody with other studies”
3. Table 4 is moved to the result section under the subheading “Comparisons of clinical characteristics of talaromycosis in patients with anti-interferon-γ autoantibody with other studies”
4. Discussion section: Lines 252-259 are removed from the manuscript.
8. Figure Files:

While revising your submission, please upload your figure files to the Preflight Analysis and Conversion Engine (PACE) digital diagnostic tool, https://pacev2.apexcovantage.com. PACE helps ensure that figures meet PLOS requirements. To use PACE, you must first register as a user. Then, login and navigate to the UPLOAD tab, where you will find detailed instructions on how to use the tool. If you encounter any issues or have any questions when using PACE, please email us at [figures@plos.org](mailto:figures@plos.org).

**Response:** We have used PACE since the last submission. If anything is incorrect, please let me know specifically what should be improved.

9. Data Requirements: Please note that, as a condition of publication, PLOS' data policy requires that you make available all data used to draw the conclusions outlined in your manuscript. Data must be deposited in an appropriate repository, included within the body of the manuscript, or uploaded as supporting information. This includes all numerical values that were used to generate graphs, histograms etc.. For an example see here: <http://www.plosbiology.org/article/info%3Adoi%2F10.1371%2Fjournal.pbio.1001908#s5>.

**Response**: As these data contains age, sex, HIV serostatus, underlying disease, and the date of events, they could be de-identified if necessary. Therefore, the dataset provided excludes the date of events and includes only 25% of the total data. If you would like to verify our analysis, the provided values should fall within the range of the complete dataset.

10. Reproducibility: To enhance the reproducibility of your results, we recommend that you deposit your laboratory protocols in protocols.io, where a protocol can be assigned its own identifier (DOI) such that it can be cited independently in the future. Additionally, PLOS ONE offers an option to publish peer-reviewed clinical study protocols. Read more information on sharing protocols at https://plos.org/protocols?utm_medium=editorial-email&utm_source=authorletters&utm_campaign=protocols

**Response**: We performed antibody testing for IFN-γ; however, we believe that a step-by-step description of the test procedure is not relevant to this study, as it falls beyond its scope. This study does not focus on the validation of the test.

11. References. Please review your reference list to ensure that it is complete and correct. If you have cited papers that have been retracted, please include the rationale for doing so in the manuscript text, or remove these references and replace them with relevant current references. Any changes to the reference list should be mentioned in the rebuttal letter that accompanies your revised manuscript. If you need to cite a retracted article, indicate the article's retracted status in the References list and also include a citation and full reference for the retraction notice.

**Response:** The reference “Nemeth E, et al.” has been removed from the manuscript in accordance with the corresponding removal of the related discussion.
